# Chicago Medical Society

**Published:** 1887-07

**Authors:** 


### CHICAGO MEDICAL SOCIETY.

[Official Report.]

STATED MEETING, April 18, 1887.

The President, W. T. Belfield, M. D., in the chair.

### PRELIMINARY DISCUSSION ON THE TREATMENT OF PULMONARY AFFECTION BY GASEOUS ENEMATA, WITH EXHIBITION ON APPARATUS.

The President: During the last twenty-five years various specifics for pulmonary tuberculosis have been announced by enthusiastic physicians, and have successively secured more or less attention from the profession, yet none of these has secured in the hands of others the results claimed by its discoverer. The benzoate of sodium treatment of Rokitansky accomplished nothing in other hands; the phenic acid injections of Declat were powerless to arrest the disease in any but his own hands; the eucalyptol treatment of Roussel failed in the hands of other investigators. The latest method of treatment—that recommended by Bergeon, of Lyons, after two years' careful observation—has, unlike its predecessors, met with almost unanimous approval at the hands of those who have tried it, including physicians in the Paris and Philadelphia hospitals and several practitioners in our own city. Since the method not only promises well, but is also universally accessible to the profession, it has been deemed advisable to present the subject to the members of the Society in a preliminary discussion this evening. There will be presented communications from Bergeon, of Lyons, Bruen, of Philadelphia, as well as the experience of several gentlemen who have used the method in this city.

Dr. Francis J. Crane read a paper entitled

BERGEON'S METHOD OF TREATING PHTHISIS BY GASEOUS ENEMATA.

After reviewing at some length this method, describing minutely the various plans that have been suggested, Dr. Crane said: I have used this treatment with four cases: two of phthisis, one of intussusception of the bowel and one of spasmodic croup. With the croup and intussusception it operated like a charm, overcoming both almost instantly. In the case of croup, I used the bi-sulphide of carbon, and in half an hour the little patient was sleeping apparently as well as ever.

*Case 1.*—Mr. W., aged 26. Two sisters and a brother died of phthisis; he had been treating with various physicians and changing climate (having been to Colorado twice) for over three years. The right lung was nearly useless, as it contained a cavity corresponding to nearly if not quite half of its original capacity. Nowhere on this side could vesicular respiration be heard, while the left apex likewise yielded unmistakable signs of disease. There were œdema of the feet, incessant cough, broken sleep, watery stools and ravenous appetite, although he could not retain anything on the stomach; temperature 102° F. After the first injection of bi-sulphide of carbon, given in the evening, he slept well for three hours and was bothered very little with cough, but on rising in the morning, to use his own words, came near strangling for want of a cough, which he finally got, and expectorated a pint with the one paroxysm. I then used the sulphuretted hydrogen water and he improved very fast; in one week he had a normal temperature; night-sweats almost entirely stopped, expectoration was much less, and he was able to wear his shoes, which he had not been able to do for over six weeks. Unfortunately, however, at the latter part of the second week he ventured out in one of our rainiest March days, took cold, and his death, two days later, cut short the record of what might have proven almost a miracle.

*Case 3.*—Mrs. W., aged 34, widow, having lost her mother and older sister from phthisis, applied to me for some heart trouble. Complained of a dizzy sensation on rising from a recumbent position; feet swollen some, hectic flush, considerable dyspnœa, slight cough with no expectoration. Diagnosis: Incipient phthisis with heart complication? She had noticed also for about a week some night-sweats, which did not last, however, after the second administration of gas. She improved so rapidly that she only made seven visits in all and pronounced herself cured. There is, however, no doubt but she will have a return of symptoms upon the slightest provocation.

This comprises all my experience, but these are facts, and facts are stubborn things to deal with. In regard to the best mineral water, I wish to say that, after trying the Lafayette, Ind., the Blue Lick, Ky., and the Ypsilanti, Mich., mineral waters, I am satisfied that the Ypsilanti mineral water is just what we want. It contains 20 cubic inches of gas to the gallon, and is so strongly impregnated with it that I use it over the second time by having solid rubber corks to replace the perforated ones when I have got through using the apparatus. Mr. St. Clair, President of the Company at 88 Randolph street, has kindly furnished a case of 12 quarts of the water, which I have forwarded to Dr. Bergeon, in hopes that it will compare favorably with the Eaux Bonnes water which he is using. I have also had an apparatus made by E. H. Sargent which I think takes the place of Dr. Morel's very well, differing from it only in point of cheapness, costing but a little more than one-half the former.

The true place of this mode of treatment cannot be established until the experience of careful observers has been given us years hence. I wish, therefore, to urge the profession to investigate the matter fairly, since time, I am confident, will prove that Dr. Bergeon has been one of the greatest benefactors of the age.

The President: This method of Bergeon has been extensively used in the Philadelphia Hospital, chiefly by Dr. Bruen, who has kindly furnished a resume of his experience with it, which the Secretary will read:

PHILADELPHIA, April 11, 1887.

The experience thus far obtained in the Philadelphia Hospital in the use of Bergeon's treatment of consumption by rectal injections of carbonic acid gas, impregnated with sulphuretted hydrogen, has been highly encouraging.

It would seem justifiable to conclude that we have a most important therapeutic agent in the gas enemata treatment. One case of acute-broncho pneumonia treated on this plan has recovered, and will be discharged from the hospital. Another case of phthisis with pneumo thorax under treatment for five weeks is so much improved that the patient insists on her discharge. The rales have disappeared from this patient's chest (the pneumo thorax was local and confined to the lower zone of right thorax), the opening into the lung has apparently closed and retraction of the chest wall is going on. This patient had been ill for a year previous to admission to the Philadelphia Hospital; the pneumo thorax was noted on admission. She has gained over nine pounds of flesh, expectoration has ceased, sweats abolished. The cough, however, continues as a modified symptom, since she is as yet merely a case of phthisis in stage of arrest, the bacilli of tuberculosis being still recognizable in the sputa.

Two cases of commencing phthisis with moist rales, dullness of percussion, harsh breathing, deficient expansion over right upper lobe, and tubercle bacilli in sputa, have recently been placed under the treatment. In these cases the gain in one week has been most material. The rales have disappeared and the patients express themselves as benefited. The method of treatment is surely worth a careful scientific investigation.

In our hands a solution of 5 grs. sulphide of sodium and 5 grs. chloride of sodium in 22 ounces of water has proven the most satisfactory substance to produce the sulphuretted hydrogen. A solution more strongly impregnated with sulphuretted hydrogen has not only been more advantageous, but seemed injurious, apparently inducing pallor, loss of appetite, and in some cases irritation of the bowels. The gas should be introduced slowly; not less than half an hour seems requisite for each seance. From three to five quarts can be introduced in most cases, a gain in the tolerance of the necessary distension of the bowel being rapidly acquired. The absorption of the gas from the bowel is very rapid and in half an hour the distension will entirely subside. It is sometimes introduced more rapidly, but the gain is in proportion to the time consumed in giving the gas.

We have not obtained as yet satisfactory results from the examination of urine, or breath, nor of the effect of the treatment upon the bacillus of tuberculosis.

EDWARD T. BRUEN, M. D.

1814 S. Rittenhouse Sq.

Dr. R. H. Babcock: My experience with this remedy is rather limited, but it seems to me there are several questions which should be considered in speaking on this subject: First, is the treatment safe? Second, is it applicable in all cases? Third, is it a cure? Practically the treatment is safe under the precautions which are laid down, but Dr. Osler, in some remarks before the Philadelphia County Medical Society, narrates a case in which the patient almost succumbed after the first treatment, the gas being charged with a mixture of bi-sulphide of carbon and sulphuret of hydrogen. We can also conceive of cases in which, on account of limited respiratory power, the rapid administration of the gas by producing distension of the large bowel would seriously embarrass the limited respiratory capacity remaining in the patient. It is also conceivable that an undue amount of distension in cases of tubercular ulceration of the bowel might be dangerous, possibly producing rupture and peritonitis; and it seems to me this is a consideration which would render us cautious about administering this treatment in typhoid fever. I have had no experience and seen no reports of cases of this disease treated in this way. In regard to its applicability in all cases, that has been partially answered in considering the previous question. It has been the experience of Geneva physicians, as reported by M. Wiss, and of De La Roche, of Lyons, that there are cases which cannot tolerate the treatment at all. One patient who had hæmorrhoids suffered from such colic that the treatment had to be abandoned. Wiss also mentions a case which the treatment has to be abandoned in the course of a month, although superintended by Bergeon himself. I was asked to administer the treatment in a case in which the lungs were so extensively destroyed that it seemed to me unwise to administer it, though perhaps a small amount of gas might have ameliorated the symptoms; but had it been possible to abate or arrest the disease, the patient could certainly not have lived with such a small amount of lung left. Of course this consideration is one which renders the indiscriminate use of the treatment hazardous. There are cases in which the recovery or arrest of the disease has been so complete that the patients are practically well and have been able to return to their avocations. However, if we are to estimate this thing by the disappearance of bacilli from the sputum we must say it is not a cure. No case is reported in which bacilli have entirely disappeared. As you know, M. Cornil has instituted a number of experiments on animals with reference to this question, a solution of which we are all looking for with much interest. French physicians are experimenting with a great many other substances.

I am now using the treatment upon two cases, one is bronchitic asthma, but the treatments are too few as yet for me to make any report. I would not wish to say whether the relief that has been obtained is due to the treatment or is merely a coincidence. Another case is a lady, aged 34, who has been suffering from phthisis for a year and a half, and who last fall received injections of phenic acid twice daily, without any improvement, who had been taking cabinet treatments from me for six weeks, without improvement, in whom the right lung is extensively consolidated, with a large cavity; in this case the relief for the first week was certainly very surprising. The patient gained in strength and experienced a feeling of well-being, or, as she expressed it, a clearer feeling in her head, a feeling of brightness which she had not had for weeks. She certainly coughed less and expectorated less at a time. Her stools had been clay-colored in spite of everything I could do; at the end of a week they were of a natural color. The movement of the bowels was perfectly natural, her tongue also cleaned off in a remarkable manner at the end of five days. The treatments were continued at my office twice a day, and the patient had to come into the city, so the improvement was perhaps not quite as great as if she had taken the treatment at home. The second week she did not do quite as well, which I attributed to the quality of the mineral water. At the end of two weeks she was improved in respect to all the symptoms which have been related this evening. The night sweats had ceased, her temperature was lower, but not normal—it ranged from 99° to 100 4-5°. I took her temperature at 9, 11, 2 and late in the afternoon, and the highest she had, with the exception of once, was 100 4-5°; it was repeatedly 98 4-5°. Her tongue did not remain clean, and her appetite did not improve as I desired. M. Bergeon states decidedly that tubercular laryngitis has been very greatly improved by this treatment. In the case just mentioned the laryngeal symptoms have improved, but whether entirely due to the treatment, I am not prepared to say, as she has had various sprays and one topical treatment to the larynx. Her case is somewhat discouraging, owing to the extent of the disease since it is not entirely confined to the right lung. She has complained of no unpleasant symptoms since the first treatment, when she complained of a slight burning at the anus and a desire to evacuate the rectum. Once or twice she complained of a little colic. She is now taking the treatments at home and feels very much encouraged, but the outlook to me is not what it should be.

I think all the precautions have been taken in this case thus far, and it proves that the treatment does not give rapid improvement in all cases, and the fact cited by Wiss, that out of four cases treated in Geneva, all died within three months, shows that it does not cure in all cases. However, it is a treatment which is so admirable and furnishes such good results in many cases that I think all physicians are justified in trying it. Another thing that recommends it is that after the patient becomes accustomed to the treatment it can be handed over to a trusty member of the family to administer. Compared with pneumatic differentiation it offers a much better chance for the improvement of the patient, although there are cases in which pneumatic differentiation will do what this will not. But thus far this method gives better prospects of benefitting a number of cases than any method which has been placed before the profession.

Dr. W. H. Weaver: A case under treatment at the Chicago Policlinic, which is making favorable progress, is one in which there is no doubt of the extent of the tuberculosis in the left upper lobe. The expectoration was quite profuse, and contained an abundance of bacilli. She has been failing for two years, but very rapidly for two months. She had fever, night sweats, prostration, wasting, loss of appetite, but had not taken any medicine of any sort. She came to the Policlinic dispensary where I treated her, the first time giving her an injection of only one pint of gas. Her temperature that day was 101½; the next day she returned and I gave her a quart injection and her temperature was then 102 6-10; the next day, the third injection, her temperature was normal and I gave her another quart. When she returned for the fourth injection her temperature was normal and she seemed better in every way; said her appetite had returned, the cough was less severe, expectoration diminished greatly, and the night sweats almost entirely relieved. At the fifth injection she had a slight temperature; she said she had had no more chills and fever for the last three days. I used a solution of about five grains of sulphide of potassium with five grains of common salt in about 20 ounces of warm water, which gives about the proper amount of sulphuretted hydrogen gas—at least the patients all say that they feel better and have no unpleasant symptoms. I have given it in three other cases: one has some pelvic trouble, and the physician who is treating her thinks that might be a counter-indication to the remedy, but she seems to be better in her general condition, has increased in flesh, and coughs less and prefers to continue the treatment. Another case of incipient tuberculosis, where the upper left apex is affected more particularly, seems to be improved considerably under treatment. He has been having the treatment one week.

Dr. Walter M. Fitch: For generating the gas we use a common Wolf bottle with three corks, one for a stop-cock for the acid, one for the delivery tube and one for the safety-valve. This last must not be pressed in too tightly; at one time I had the stop-cock blown out and broken by putting in the acid a little too rapidly. To make the gas we use about a heaping tablespoon of bicarbonate of sodium, covering that with cold water about two inches deep. If we use hot water we make a suds that will foam up and run into the bag. We use the bicarbonate of sodium and sulphuric acid diluted one part in four. The reason for using the sulphuric acid in preference to any other is that, being absolutely non-volatile, none of it will pass over into the bag of carbonic acid gas. To make the gas the generating bottle is connected with the bag, which is emptied and tightly rolled in order to remove all the air. The generator should also be emptied of air by turning in some of the acid before it is connected with the gas-bag. The bags usually hold about one-half of what they are marked—a three-gallon bag will hold about six quarts, etc. If we should over-distend it the corks would be blown out of the generator. For injecting the gas the stop-cock is turned off and the bag is connected with the bubbling-tube, which extends to the bottom of the bottle of mineral water, so that the gas coming from the bag may pass down into the water, and rising up through it be medicated by the sulphur vapor which it contains. It is injected by means of a hand-bulb. In using the bulb it is important to remember that since it is intended to be used only with water, the action of the valves is not perfect with gas, and it is therefore necessary that the bulb be held vertically, so that the weight of the valves may assist in closing them. In using it in this way we secure a perfect action which draws the gas into the bottom of the bottle and delivers it through the tube. The bisulphide of carbon has been mentioned several times. In order to use this it is necessary that the gas be washed for fear that it may contain impurities, for none of the chemicals we get are perfectly pure, and as they are most likely to contain vapors of hydrochloric acid which are easily dissolved in water, the gas is bubbled through pure water to purify it. Between the Wolf bottle and the bulb is inserted an ordinary chemical drying-tube, a glass tube three-fourths of an inch in diameter, which is drawn down at one end small enough to have the rubber tube slipped over it. Into the other end is fitted a cork with a glass tube through it; in the drying-tube is placed a tampon of cotton, and the requisite amount, usually one drachm, of bisulphide of carbon is placed on the cotton. In passing the gas through this cotton it becomes very strongly impregnated with the vapor and is injected in that manner. I think some Philadelphia physician spoke of an instrument for measuring the gas injected. A very simple way of measuring it when using a small apparatus of this kind is with the bulb. The bulb will deliver at each compression just so much gas; in order to measure how much is delivered we simply take a measured bottle, invert it over the water with the water filling it, then introduce the rectal tube into the bottle and remove the water from it by displacement, the number of compressions being counted. The bulbs vary in size. I remember the capacity of six now in use: two count 15 compressions to the pint, three count 20 compressions to the pint, and one counts 25. Thus, with each apparatus the bulb must be measured, and knowing its capacity, the gas can be prescribed in as definite a way as any medicine measured out of a bottle.

The President: My experience with this method, extending over less than a month, comprises the observation of one case that impressed me strongly; this was a young man suffering from the last stages of quick consumption, who had been in the care of Dr. H. A. Johnson. He was very feeble, much emaciated, had badly swollen feet and albuminous urine. His temperature ranged from 101 to 103 each afternoon, and he had profuse night sweats. In addition to these usual symptoms he suffered to an extraordinary degree from laryngeal tuberculosis. He had been unable for many weeks to lie down with comfort, and according to his own statement had not slept fifteen minutes in three months. Cough was constant and expectoration profuse. About a month before Dr. Johnson had given him three or four weeks to live. I used gaseous enemata, employing the artificial solution advised by Dr. Bruen, twice a day. Improvement began on the second day, and by the fourth day was pronounced. The cough was less distressing and the sweats and expctoration had diminished; the patient was able to lie down and sleep for two hours continuously. He was, however, so evidently near dissolution that I deemed it inadvisable to continue a tentative treatment, and stopped it on the fourth day. Two days later he sent an urgent request for the resumption of the treatment, saying that his former symptoms were returning. However, I declined to use the injections further, and six days after their discontinuance the patient died, nearly two weeks after his physician had expected his demise.

It should be remembered that these injections seem to arrest, not the tuberculosis proper, but the sepsis, which is secondary thereto, and that there are numerous other septic conditions in which benefit may fairly be expected, such as surgical sepsis, diphtheria, puerperal fever, etc. The apparatus furnished by the stores is unnecessarily expensive and inconvenient for a visiting practice. I use an improvised apparatus consisting of a gas-bag, double perforated cork and ordinary large, wide-mouthed bottle—the whole costing $2.50 instead of $10. The entire outfit can be readily improvised at a little cost and carried in a small hand-bag.

My limited experience gives me the impression that the artificial solution of the sulphide of potassium is preferable to either the Ypsilanti or the Blue Lick water, because the amount of sulphuretted hydrogen in these waters, as we obtain them at the drug store, appears to be extremely variable, while in the artificial solution it can be maintained at a given percentage, increased or diminished as desired, yet care should be taken to make a weak solution, about such as is recommended by Dr. Bruen, since a too concentrated solution of the poison may produce very unpleasant symptoms. This method can be employed by every practitioner as well as by the most expert specialist. If it will accomplish nothing more than the relief of the distressing cough, expectoration, fever and sweats of advanced consumption, it will at least do what can be effected by no other therapeutic measure with which I am acquainted; hence it is to be hoped that it will be submitted to a general test by the profession, that we may quickly ascertain exactly its merits and demerits.

Dr. C. M. Fitch: The remedies that have been offered for consumption in the past are about as numerous as the sure-cures that have been offered for cancer and hydrophobia, and have commanded about as much attention from the profession. But this method of Bergeon's comes to us under very different auspices; it comes with a letter of introduction that demands from us respectful attention. J. Henry Bennett endorses Bergeon as a man worthy of credit; and if Bergeon's statements be true, they are of the deepest interest to us all. It is in Bergeon's favor also that he not only asks us to believe something, but he gives us a reason for so believing—a plausible explanation of what he wishes us to receive. He assumes that septicæmia, arising either from the absorption of pus, or the morbid product of the bacilli, is the cause of the formidable symptoms of phthisis, and that if we can antagonize this septicæmia, can undo day by day the deadly work of the bacilli, we shall gain time in which the lesions that have arisen from phthisis may be overcome.

The first case in which I had occasion to test this method of Bergeon was one of extreme anxiety to me. The patient, a young lady 20 years of age, had previously had exceptionally good health, was well nourished, with a muscular development that seemed to defy fatigue. She returned in July last from a visit to Colorado with a cough which was referred to the dryness of the climate and the inhalation of the alkaline dust, which causes a catarrhal irritation. This seemed a plausible explanation, and as the cough was not urgent, it received but little attention until, in November, she took a cold which resulted in chills for three or four successive days, and seemed to prostrate her greatly. I then examined her chest, and to my astonishment found extensive and marked consolidation at the apex of the left lung; there was also irritation throughout the remainder of the lung and some moist râles. I was much alarmed by what I found, for the location of the consolidation made me fear it might prove something more than simple pneumonia. Several of our best physicians tendered their aid, but the case failed to improve. The patient lost flesh rapidly, her cough became fearfully oppressive and her temperature never fell to normal, rarely below 100°, and most of the time 101° and rising to 103°. She had daily chills and profuse night-sweats, and the case presented all the features of acute phthisis. At the last examination, made by Dr. Belfield and Dr. Ochsner, both thought they detected bacilli in the sputum, although previous examinations had failed to show them.

Late in January my attention was called to an article in the *Medical Record*, giving a notice of Bennett's paper in the *British Medical Journal*, which contained an account of Bergeon's method. After considerable trouble I obtained a copy of the paper which contained this article, and went to Sargent's to have an apparatus made, and there learned that they had just made one for Dr. Crane, who kindly allowed me to appropriate it, and ordered another for himself. Knowing nothing at that time of what we might expect in such a case from our different mineral waters, and knowing that Dr. Bergeon recommended bi-sulphide of carbon as the best substitute, we commenced treatment with the bi-sulphide and the effect was surprising. The treatment was commenced seven weeks ago to-day; during the week previous the temperature had at no time fallen to normal; the lowest point was 100°, and once it rose to 105½°, and night-sweats and chills were constant. The first time the injection was tried very cautiously, only a moderate amount of gas being used. The effect was not specially apparent; the second day the temperature still rose to 103°. That night the gas was used much more freely and with results that to us were somewhat alarming. The next morning the temperature fell to 95°, and it did not rise above 100° during the day; the cough was almost suppressed, although it had previously been very distressing. The next day we did not use the gas quite so freely, but the day following the lung was almost entirely occluded; the mucus seemed to become viscid, and the air was almost shut out from the lung. There was so little elimination of the bi-sulphide through the lung that we soon began to have marked symptoms of bi-sulphide poisoning, although in many respects she appeared better; the cough was almost gone, and there were no chills. The treatment was discontinued for about ten days, and then resumed with the Ypsilanti mineral water. From that time there has been a satisfactory improvement, and she is better than she has been at any time since her sickness began; her appetite and strength are improved, and she is evidently beginning to gain flesh; her cough is greatly lessened and the chills have altogether disappeared. Her night sweats have been absent for six weeks; although during the last warm weather she perspired a little for three or four nights, there was nothing in the nature of colliquative sweating, and she seems now in a fair way to recover.

